# Polygenic risk score, healthy lifestyles, and risk of incident depression

**DOI:** 10.1038/s41398-021-01306-w

**Published:** 2021-03-29

**Authors:** Zhi Cao, Hongxi Yang, Yixuan Ye, Yuan Zhang, Shu Li, Hongyu Zhao, Yaogang Wang

**Affiliations:** 1 School of Public Health, Tianjin Medical University, Tianjin, China; 2 Department of Big Data in Health Science, School of Public Health, Zhejiang University School of Medicine, Hangzhou, China; 3 Department of Biostatistics, School of Public Health, Yale University, New Haven, CT USA

**Keywords:** Depression, Clinical genetics

## Abstract

Genetic factors increase the risk of depression, but the extent to which this can be offset by modifiable lifestyle factors is unknown. We investigated whether a combination of healthy lifestyles is associated with lower risk of depression regardless of genetic risk. Data were obtained from the UK Biobank and consisted of 339,767 participants (37–73 years old) without depression between 2006 and 2010. Genetic risk was categorized as low, intermediate, or high according to polygenic risk score for depression. A combination of healthy lifestyles factors—including no current smoking, regular physical activity, a healthy diet, moderate alcohol intake and a body mass index <30 kg/m^2^—was categorized into favorable, intermediate, and unfavorable lifestyles. The risk of depression was 22% higher among those at high genetic risk compared with those at low genetic risk (HR = 1.22, 95% CI: 1.14–1.30). Participants with high genetic risk and unfavorable lifestyle had a more than two-fold risk of incident depression compared with low genetic risk and favorable lifestyle (HR = 2.18, 95% CI: 1.84–2.58). There was no significant interaction between genetic risk and lifestyle factors (*P* for interaction = 0.69). Among participants at high genetic risk, a favorable lifestyle was associated with nearly 50% lower relative risk of depression than an unfavorable lifestyle (HR = 0.51, 95% CI: 0.43–0.60). We concluded that genetic and lifestyle factors were independently associated with risk of incident depression. Adherence to healthy lifestyles may lower the risk of depression regardless of genetic risk.

## Introduction

Depression is a major cause of death in the UK population, and incidence rate has been on the rise, as 19.7% UK people aged 16 years and older showed symptoms of depression or anxiety in 2014^1^. According to data from the 2014 National Health Service (NHS), 39% of individuals with a diagnosis of depression or anxiety were taking related medications^2^. Depression is a complex disease that is affected by both genetic and environmental factors. The effect of genetic predisposition on the risk of depression has been examined in the previous studies^3,4^. A meta-analysis showed that the heritability for depression was 37% (95% CI: 31–42%), and data from family studies have reported a two- to three-fold increase in the risk of depression in the first-degree offspring of patients with depression^5^. In the last ten years, several genome-wide association studies (GWASs) have identified polymorphisms associated with the development of depression^6–8^. These single nucleotide polymorphisms (SNPs) provided quantitative measures of genetic susceptibilities and may be predictive of the incidence of depression^9^.

Lifestyle is a common and crucial modifiable risk factor for depression. A large number of evidence has established that many individual lifestyle factors—such as non-smoking^10^, a healthy diet^11^, physical activity^12^, moderate alcohol intake^13^, and a healthy weight^14^—are associated with decreased risk of depression. However, because many of the lifestyle factors often coexist, exploring the joint impact of these lifestyle behaviors on the risk of depression is extremely relevant. Yet, there is limited study on this topic. As for other diseases, recent studies have shown that adherence to a combined healthy lifestyles plays a key role in attenuating the impacts of genetic risks of several chronic diseases, including coronary heart disease^15^, stroke^16^, hypertension^17^, diabetes^18^, dementia^19,20^, breast cancer^21^, and colorectal cancer^22,23^. However, to date, no studies have examined whether an advantageous combined lifestyle factors attenuate on the risk of developing depression.

Therefore, in the present study, to further evaluate depression risk and lifestyle factors, we created a combination of healthy lifestyle scores based on recommendations of five potentially modifiable lifestyle factors: smoking, diet, physical activity, alcohol intake, and body fat. Our present study included the following aims: (1) to explore the associations of genetic risk and combined lifestyle factors with the risk of incident depression; and (2) to investigate whether adherence to a healthy lifestyle might counteract genetic risk for depression, using data from a large-scale cohort of the UK population.

## Methods

### Study design and population

Our study derived from UK Biobank, a prospective population-based cohort study. The UK Biobank recruited 502,528 adults (37–73 years old) from the general population between 2006 and 2010. Participants came from 22 assessment centers across England, Scotland, and Wales, where they completed touchscreen and nurse-led questionnaires, had physical measurements taken, and provided biological samples^24^. The exposures of interest in our study included imputed genetic data and lifestyle (alcohol intake, smoking, diet, body mass index [BMI], and physical activity). In the current study, we included all participants who were classified as white British and without a history of depression that were self-reported and/or medical records, and with complete data on their genetics and lifestyle factors.

### Lifestyle factors

We defined the following five healthy lifestyle factors on basis of the American Heart Association (AHA) guidelines: moderate alcohol intake, no current smoking; healthy diet; BMI < 30 kg/m^2^ and regular physical activity^16,25^.

In our study, a healthy diet was determined according to the increased consumption of fruit, vegetables, and fish, as well as the decreased consumption of processed meats and red meats. A healthy diet was defined if a individual meets at least two of the healthy food items listed above. Supplemental text S1 provides additional details on our construction of a healthy diet score. Moderate alcohol intake was defined as 0–28 g/d for men and 0–4 g/d for women. Moderate physical activity was defined as at least 150 min of moderate-intensity activity weekly or 75 min of vigorous activity weekly.

Participants scored 1 point if they meet an healthy lifestyle factor. The total lifestyle scores ranged from 0 to 5, with higher scores meaning a healthier lifestyle. Lifestyle behaviors were further categorized as favorable (4 or 5 healthy lifestyle factors), intermediate (2 or 3), and unfavorable (0 or 1) lifestyles. Additionally, a weighted-standardized healthy lifestyle score was established based on the β coefficient of each lifestyle factor in the Cox model with all five lifestyle factors, as well as adjustments for other covariates. The initial dichotomy lifestyle factors were multiplied by the β coefficients and were then summed, divided by the sum of the β coefficients, and multiplied by 100. According to the distribution of unweighted lifestyle scores, the weighted-standardized lifestyle scores were grouped by favorable, intermediate, and unfavorable^26^.

### Polygenic risk score

Polygenic risk score (PRS) was constructed to assess the cumulative genetic risk for depression. The score was constructed based on the GWAS summary-statistic data of depression in the Genetic Epidemiology Research on Adult Health and Aging (GERA)^27^. The summary-statistic data were based on people of European ancestry; therefore, the present study was restricted to European samples in the UK biobank. The posterior effect sizes of SNPs on depression were inferred using PRS-CS, a polygenic prediction method^28^. An individual-level polygenic score was defined as the sum of the number of risk alleles present at each SNP weighted by the corresponding posterior effect sizes across all available SNPs (*N* = 1,065,182) in the UK biobank, and it was produced using the plink “–score” command. The PRS for all individuals in UK Biobank were z-standardized and then categorized into low, intermediate, and high risks.

### Incidence of depression

Depression was ascertained using hospital inpatient records containing data on admissions and diagnoses. Participants were followed up until the date of incident depression, death or end of study (March 31, 2017). Diagnoses were recorded using the International Classification of Diseases (version 10; code ICD-10) coding system. Participants with depression were identified as having a primary/secondary diagnosis (hospital records) using ICD-10 codes.

### Covariates

All analyses were adjusted for age, sex, qualifications (college degree; A levels/AS levels, O levels/General Certificate of Secondary Education (GCESs); Certificate of Secondary Education (CSEs); National Vocational Qualification (NVQ); Higher National Diploma (HND) or Higher National Certificate (HNC); other professional qualifications), socioeconomic status (categories derived from Townsend deprivation index, combining information on social class, employment, car availability, and housing), and the first ten principal components of ancestry.

### Statistical analyses

Baseline characteristics of the participants were summarized across depression status as the percentage for categorical variables and the mean and standard deviation (SD) for continuous variables. Person-year were calculated from the date of recruitment to date of developing depression or censoring time (whichever event occurred first). Incident rates and 95% confidence intervals (95% CIs) per 1000 person-years were estimated by the exact Poisson test, and analyses were stratified by genetic risk.

Cox proportional hazard regression models were utilized to explore the association of PRS, lifestyle, and combined genetic and lifestyle categories with risk of depression. The proportional hazard assumption was checked by tests based on Schoenfeld residuals, and the results suggested that the assumptions had not been violated (Supplemental Fig. S1). The primary model was adjusted for age, sex, socioeconomic status, qualifications, and the first 10 principal components of ancestry. Then, we fitted an additional model such that PRS and lifestyle category were adjusted for one another. To further investigate whether and to what extent healthy lifestyle practices may counteract the harmful effect of high genetic risk on incident depression, the associations between lifestyle factors and depression stratified by genetic risk were explored. An interaction term in the Cox model was included to test for a statistical interaction between lifestyle and genetic risk in relation to depression.

### Sensitivity analysis

We undertook a series of analyses to evaluate the robustness of our findings. First, the risk of depression was examined by using a weighted lifestyle score. Second, potential differences in risk of depression were explored by sociodemographic factors in subgroup analyses, including age (middle-aged [37–59 years old] or older adults [≥60 years old]), sex (male, female) and socioeconomic status (low, intermediate, high). Third, to minimize the possibility of spurious associations due to reverse causation, the associations of genetic risk and lifestyle with depression were re-analyzed after excluding the people who died during the first two years of follow-up. Fourth, waist-to-hip ratio (WHR) is often used as an indicator of body fat, BMI was replaced with WHR to include the lifestyle criteria. Fifth, a competing risk regression was also fitted using the sub-distribution method proposed by Fine and Grey, with the competing event being the death from all causes other than developing depression. Finally, E-value was calculated to assess the effect of unmeasured confounding. The E-value evaluates the minimum strength that an unmeasured confounding variable that must offset the observed association of exposure with outcomes when considering all other measured covariates^29^. The E-value contributed to evaluate the plausibility of unmeasured confounding and towards the evidence for causality^30^.

To maximize the likelihood of reporting true findings, we set the *α* at 0.05 and used Bonferroni correction to adjust for multiple testing. We considered two -sided *P* values less than 0.05 (*P* value of less than 0.05 divided by the number of tests, i.e., 0.05/3) statistically significant. All analyses were performed using STATA 15 statistical software (StataCorp).

## Results

**Fig. 1:**
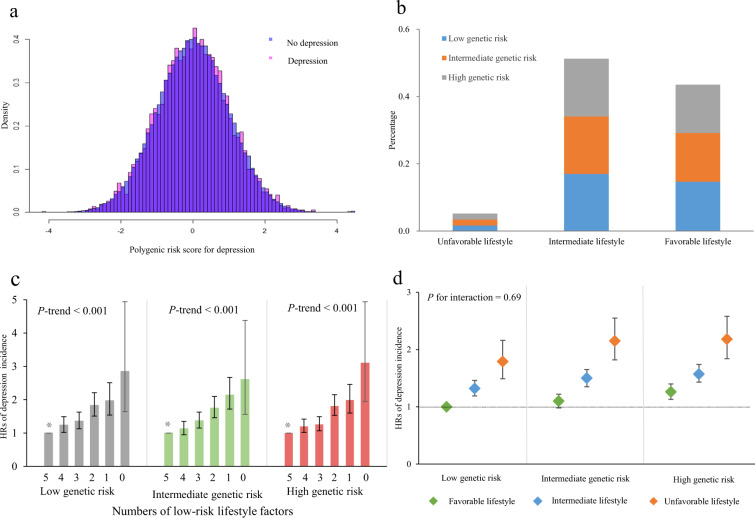
Risk of incident depression according to combined genetic risk and lifestyle factors profile. **a** Distribution of PRS by incident depression; **b** percentage of genetic risk according to lifestyle factors categories; **c** numbers of healthy lifestyle factors and depression according to genetic risk categories; **d** the risk of combined genetic risk and lifestyle factors profile for incident depression.

At baseline, 502,528 participants were assessed. After excluding non-White participants (*n* = 29,811) with prevalent depression (*n* = 29,239), as well as those without genetic information (*n* = 12,709) or lifestyle information (*n* = 90,648), a total of 339,767 participants was finally included in our study. Baseline characteristics of the participants are provided in Table 1. During a total of 2,722,320 person-year (a median follow-up of 8.1 years), there were 5739 cases of developing depression. PRS was normally distributed (Fig. 1a), and almost half of participants engaged in 4 or 5 (43.6%) of the 5 healthy lifestyle factors (Fig. 1b).

**Table 1:** Characteristics of participants at baseline.

Characteristics	All (*N* = 339,767)	Incident depression (*N* = 5,739)	No depression (*N* = 334,028)	*P* value
Age, mean (SD)	56.6 (8.1)	56.8 (8.2)	56.6 (8.1)	0.176
Sex				<0.001
Male	161,463 (47.5)	2229 (38.8)	159,234 (47.7)	
Female	178,304 (52.5)	3510 (61.2)	174,794 (52.3)	
Townsend deprivation index, mean (SD)	−1.62 (2.89)	−0.85 (3.25)	−1.63 (2.88)	<0.001
Qualifications				<0.001
College degree	118,288 (34.8)	1417 (24.7)	116,871 (35.0)	
A levels/AS levels	39,459 (11.6)	587 (10.2)	38,872 (11.6)	
O levels/GCESs	72,547 (21.4)	1,293 (22.5)	71,254 (21.3)	
CSEs	17,629 (5.19)	413 (7.2)	17,216 (5.2)	
NVQ or HND or HNC	22.505 (6.6)	426 (7.4)	22,079 (6.6)	
Other	17,475 (5.1)	322 (5.6)	17,153 (5.1)	
None of the above	49,616 (14.6)	1228 (21.4)	48,388 (14.5)	
Healthy lifestyle factors				
No current smoking	308,615 (90.8)	4776 (83.2)	303,839 (90.9)	<0.001
Healthy diet	149,811 (44.1)	2531 (44.1)	147,280 (44.1)	0.988
BMI < 30 kg/m^2^	266,971 (78.6)	3971 (69.2)	263,000 (78.7)	<0.001
Regular physical activity	211,798 (62.3)	3453 (60.2)	208,345 (62.4)	0.001
Moderate alcohol intake	174,359 (51.3)	2535 (44.2)	17,1824 (51.4)	<0.001
Healthy lifestyle scores				<0.001
0 or 1 healthy lifestyle	17,655 (5.2)	498 (8.7)	17,157 (5.1)	
2 or 3 healthy lifestyles	174,175 (51.3)	3238 (56.4)	170,937 (51.2)	
4 or 5 healthy lifestyles	147,937 (43.5)	2003 (34.9)	145,934 (43.7)	
Genetic risk category				<0.001
Low	113,333 (33.4)	1707 (29.7)	111,626 (33.4)	
Intermediate	113,197 (33.3)	1931 (33.7)	111,266 (33.3)	
High	113,237 (33.3)	2101 (36.6)	111,136 (33.3)	

Values are numbers (participants) unless stated otherwise.

Table 2 presents the incidence rates and HRs of depression across genetic risk categories. The incidence rates per 1000 person-years were 1.88 (1.79–1.97), 2.13 (2.04–2.23), and 2.32 (2.22–2.42) in participants with low, intermediate, and high genetic risk, respectively. The cumulative incidence rate of depression raised with increasing genetic risk category (log-rank test <0.001) (Supplementary Fig. S2). The risk of incident depression also increased monotonically across genetic risk categories (*P* for trend < 0.001). The HRs of depression were 1.13 (95% CI: 1.06–1.20) and 1.22 (95% CI: 1.14–1.30) for intermediate and high genetic risk categories compared to those with low genetic risk after adjustment for potential confounders. Additional adjustments for lifestyle factors did not essentially change these results, indicating that genetic risk for depression was statistically independent of lifestyle factors. Restricted spline curve showed a linear and dose–response association between PRS and depression (*P* for non-linear = 0.395), with HR being 1.09 (95% CI: 1.06–1.12) per 1-SD increment (Supplementary Fig. S3).

**Table 2:** Risk of incident depression according to genetic risk and lifestyle categories.

	Events/person-year	Incidence rate per 1000 person-year	Model 1^a^	Model 2^b^
Genetic risk				
Low	1707/908,528	1.88 (1.79–1.97)	1 (Ref.)	1 (Ref.)
Intermediate	1931/906,595	2.13 (2.04–2.23)	1.13 (1.06–1.20)	1.12 (1.05–1.20)
High	2101/907,197	2.32 (2.22–2.42)	1.22 (1.14–1.30)	1.22 (1.14–1.30)
*P* for trend^c^			<0.001	<0.001
Lifestyle categories				
Favorable	2003/1182,008	1.69 (1.62–1.77)	1 (Ref.)	1 (Ref.)
Intermediate	3238/1398,655	2.32 (2.24–2.40)	1.30 (1.23–1.38)	1.31 (1.24–1.39)
Unfavorable	498/141,658	3.52 (3.22–3.84)	1.81 (1.64–2.00)	1.83 (1.65–2.02)
*P* for trend^c^			<0.001	<0.001

^a^Model 1 was adjusted for age, sex, qualifications, socioeconomic status and first 10 principal components of ancestry.

^b^Model 2 was adjusted for Model 1 and lifestyle categories (for genetic risk model) and genetic risk (for lifestyle factors model).

^c^*P* value for trend was calculated treating the genetic risk score or lifestyle factors as a continuous variable.

The incidence rate and risk of depression also increased monotonically across lifestyle categories (Table 2). Compared with a favorable lifestyle, an unfavorable lifestyle was associated with a high risk of depression (HR = 1.81, 95% CI: 1.64–2.00). Likewise, additional adjustment for genetic risk resulted in an HR of 1.83 (95% CI: 1.65–2.02), in line with the independence of genetic and lifestyle factors. A risk gradient was noted across the number of healthy lifestyle factors, such that the participants with no healthy lifestyle were at significantly higher risk of depression than those with five healthy lifestyle factors (Supplemental Table S1). Moreover, there were a dose–response relationship between the increasing lifestyle factors and depression, regardless of genetic risk (all *P* for trend <0.001) (Fig. 1c). In addition, we calculated correlations between individual lifestyle factors and found only weak correlations (*r* < 0.1) (Supplemental Table S2). WHR, rather than BMI, was included in lifestyle factors, the association between lifestyle and depression was not essentially changed (Supplemental Table S3).

In both sex and age subgroup, the effect of high genetic risk on depression was stronger in female than male. Results were consistent in that genetic risk was monotonically associated with the risk of depression in subgroups of age. A monotonic association between lifestyle and depression was also observed for both sex and age subgroups (Supplemental Table S4). The modification effect of socioeconomic status on the association of lifestyle and genetic risk with depression were also explored, we found that high genetic risk was not significantly associated with depression in high socioeconomic status category, the beneficial effect of lifestyle on depression was stronger in high socioeconomic status than lower socioeconomic status (*P* for interaction <0.05) (Supplemental Table S5).

When we conducted analyses of combined genetic risk and lifestyle categories, there was an overall monotonic association with an increasing genetic risk and an unfavorable lifestyle (Fig. 1d). People with a high genetic risk and an unfavorable lifestyle had a highest risk of depression compared with participants with low genetic risk and a favorable lifestyle (HR = 2.18, 95% CI: 1.84–2.58). No significant interaction between lifestyle and genetic risk in relation to depression was observed (*P* for interaction = 0.69) (Supplemental Table S6), meaning that the association with lifestyle factors did not vary substantially across genetic risk categories. Sensitivity analyses showed similar results among participants with at least two years of follow-ups (Supplemental Table S7). The competing risk analysis was also in accordance with the main analysis on the association of combined genetic risk and lifestyle factors with the incident depression (Supplementary Table S8).

**Fig. 2:**
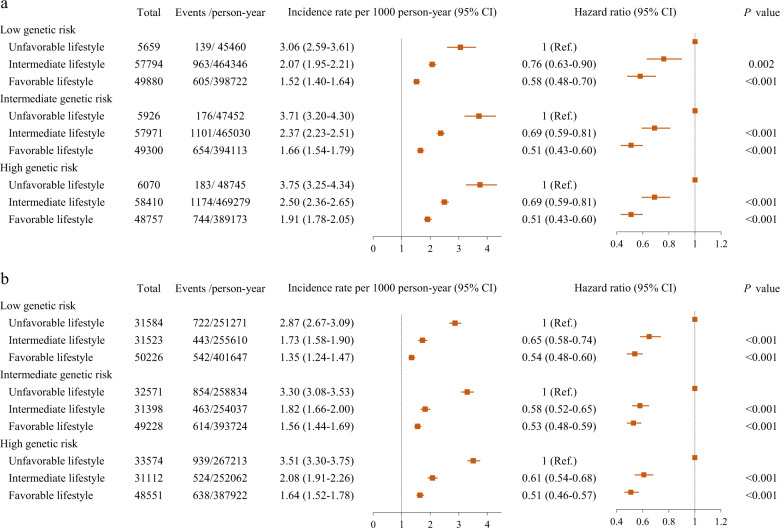
The beneficial effect of healthy lifestyle factors on the risk of incident depression stratified by genetic risk profile. **a** Unweight lifestyle score and depression according to genetic risk; **b** weight lifestyle score and depression according to genetic risk.

Further analyses stratified by genetic risk category, confirmed that a favorable lifestyle was related to a lower risk of depression across genetic risk categories (Fig. 2a). Among participants who had a low genetic risk of depression, intermediate and favorable lifestyles were associated with 24% (HR = 0.76, 95% CI: 0.63–0.90) and 42% (HR = 0.58, 95% CI: 0.48–0.70) lower risks of depression, respectively. Among those at high genetic risk, intermediate and favorable lifestyles separately reduced the risk of depression by 31% (HR = 0.69, 95% CI: 0.59–0.81) and 49% (HR = 0.51, 95% CI: 0.43–0.60), respectively. These results were not essentially altered much when weight lifestyle scores were used (Fig. 2b). In addition, among individual components of lifestyle factors, no current smoking and non-obesity contributed most to reduce the risk of developing depression, regardless of genetic risk (Table 3). The E-values analysis showed that the point estimate and lower confidence bound were 1.74 and 1.54 for high genetic risk, 3.06 and 2.69 for unfavorable lifestyle, respectively. To offset the observed association, an unmeasured confounding variable must be correlated with both exposure and outcomes, at least the risk ratio of the E-value at each time point, and conditional on other confounding variables.

**Table 3:** Multivariable Cox regression analysis of individual lifestyle factors in relation to risk of depression, stratified by genetic risk.

Lifestyle factors	Low genetic risk	Intermediate genetic risk	High genetic risk
No current smoking	0.53 (0.47–0.61)	0.54 (0.48–0.62)	0.56 (0.49–0.63)
BMI < 30 kg/m^2^	0.69 (0.62–0.76)	0.66 (0.60–0.73)	0.63 (0.57–0.69)
Healthy diet	1.02 (0.93–1.13)	0.97 (0.89–1.07)	1.07 (0.98–1.17)
Regular physical activity	0.88 (0.80–0.97)	0.90 (0.82–0.98)	0.95 (0.87–1.04)
Moderate alcohol intake	0.93 (0.85–1.03)	0.92 (0.84–1.00)	0.85 (0.78–0.93)

The analyses were adjusted for age, sex, qualifications, socioeconomic status and first 10 principal components of ancestry, individual lifestyle factor was adjusted for each other.

## Discussion

In this large-scale prospective study, we found that genetic risk and lifestyle factors were independently associated with the risk of depression. Participants with high genetic risk and unfavorable lifestyle profile had a more than two-fold increased risk of incident depression compared with that of participants with a low genetic risk of depression and a favorable lifestyle. No significant interaction between genetic risk and healthy lifestyle was observed. The beneficial impact of healthy lifestyle on depression was found across all genetic risk categories, suggesting that the genetically predetermined rise in depression can be counteracted, at least to some extent, by a healthy lifestyle. These findings provide evidence for the necessity of a combination of healthy lifestyle factors in preventing depression and reinforce the considerable potential of primary prevention.

Our present study adds to previously reported studies on individual lifestyle factors and risk of depression. Although previous studies found that individual lifestyle factor, such as non-smoking^10^, a healthy diet^11^, physical activity^12^, moderate alcohol intake^13^, and a healthy weight^14^, was related to depression, the samples of these studies were relatively smaller. Moreover, our present study is the first to incorporate information of PRS. Recently, GWASs have identified several common genetic loci that are significantly related to risk of depression^31,32^. The contribution of an inherited genetic predisposition to the occurrence of sporadic depression has also previously been reported. A clinical and epidemiological study of a youth cohort suggested that PRS may serve as an early indicator of clinically significant levels of depression^9^. A recent study also showed that PRS was associated with depression^33^. Similar to this previous study, our present study demonstrated that a higher genetic risk increased the risk of depression by 22%.

A reduced risk of depression by adherence to a healthy lifestyle have been well documented^34,35^. To the best of our knowledge, however, only a limited number of studies have examined the associations of combined healthy lifestyle factors with risk of developing depression in a general UK population. The risk reduction linked to healthy lifestyle in the current study was consistent across all strata of genetic risks, which emphasizes the benefits for entire populations in adherence to a healthy lifestyle, regardless of genetic risk. Among these lifestyle factors, the most statistically significant associations were discovered for no current smoking and for a BMI < 30 kg/m^2^. These findings further supplemented the current recommendation that people with high genetic predisposition of depression should clinically practice a more healthier lifestyle factors to maintain a lower risk of developing depression.

The precise mechanisms by which the combination of lifestyle and genetic factors affect the risk for depression remain unclear. The plausible biological effects of each factor are pleiotropic in nature and likely involve overlapping influences via pathways that were related to the development of depression. Common genetic variants related to depression might have an effect on both personality and psychological regulation^36^. Both neuroendocrine disruption and inflammation have also been associated with the etiology of depression. Obesity, poor diet, exposure to chemicals or pollutants, and high stress levels may potentially break the hypothalamic-pituitary-adrenal axis and increase cortisol, low-grade systemic inflammation, and oxidative stress^36,37^. Although the judicious use of medications and psychological techniques was recommended, the emerging evidence encouraged a more integrative approach for managing depression and acknowledged that modifiable risk factors, such as lifestyle intervention, should be a routine part for treatments and preventative efforts.

### Strengths and limitations

The strengths of our present study included its large sample size of UK Biobank participants, its prospective design, information on a wide-range of depression-associated SNPs and the use of standardized protocols for data collection. Another distinctive feature of our study compared with prior studies is that all SNPs associated with depression were included to calculate PRS. Despite these strengths, several limitations of the current study need to be considered. First, behavioral changes before or after baseline examinations may affect our results. Second, although all models were adjusted for known potential biases and participants were followed up for a median of 8.1 years, it is possible that unmeasured confounds and reverse causation remained. Third, depression was diagnosed by hospital inpatient records in our study, although diagnosed by a doctor is a more common and precise way. Milder depression or severely depressed people who very hardly go to the hospital unless they are in danger of life may easily be underestimated. Finally, this study was primarily restricted to participants of European ancestry aged 37–73 years at baseline and, therefore, further research is warranted to examine to what degree these results generalize to other populations.

### Implications for further research

We found that healthy lifestyle factors were associated with a substantially lower risk of depression regardless of genetic risk, suggesting that genetic risk might be attenuated by adherence to a healthy lifestyle. Our results provide encouraging support of lifestyle recommendations for depression prevention that highlight a combination of healthy lifestyle factors for everyone. From a public health viewpoint, our findings should be used to strengthen the importance of primary prevention to policymakers, as well as to support population-wide efforts to lower the risk of incident depression through combined lifestyle modifications. Future research is needed to investigate the potential mechanism of depression in relation to lifestyle factors and genetic risk.

## Supplementary information

Supplemental Marterial

## Data Availability

The data that support the findings of this study are available from UK Biobank project site, subject to successful registration and application process. Further details can be found at https://www.ukbiobank.ac.uk/.
