# Supplementary material for: Polygenic risk score, healthy lifestyles, and risk of incident depression
Source: Transl Psychiatry. 2021 Mar 29;11:189. doi: 10.1038/s41398-021-01306-w (PMC8007584; doi:10.1038/s41398-021-01306-w)
Supplement: Supplementary file 1 — Supplemental Marterial [file 41398_2021_1306_MOESM1_ESM.doc]

**Supplemental materials**

Text S1 Diet score extended information

During the baseline assessment all UK Biobank participants completed an extensive questionnaire that included dietary habits (“Touchscreen Questionnaire”, available from http://www.ukbiobank.ac.uk/resources/).

Healthy diet patterns were adapted from the American Heart Association Guidelines

and defined as follows:

Total fruit and vegetable intake: > 4.5 pieces or servings a week. 3 Tablespoons of

vegetables were considered one serving

Total fish intake: > 2 per week

Processed and red meat intake: 2 or fewer times intake of processed meat per week &

5 or fewer times intake of red meat per week

The healthy diet score was dichotomised as 1 = at least two of the healthy food items,

0 = fewer than 2 of the healthy food items.

Table S1: Risk of Incident depression According to Healthy Lifestyle scores.

| Healthy lifestyle scores | HR (95% CI) | P value |
| --- | --- | --- |
| 5 healthy lifestyle factors | 1 (Ref.) |  |
| 4 healthy lifestyle factors | 1.19 (1.07-1.32) | 0.001 |
| 3 healthy lifestyle factors | 1.32 (1.20-1.47) | <0.001 |
| 2 healthy lifestyle factors | 1.80 (1.62-1.99) | <0.001 |
| 1 healthy lifestyle factor | 2.04 (1.79-2.32) | <0.001 |
| 0 healthy lifestyle factor | 2.85 (2.13-3.82) | <0.001 |
| *P* for trend | <0.001 | |

Table S2: Correlations between individual lifestyle factors

| Pearson correlation coefficient | No current smoking | BMI <30 kg/m^2^ | Healthy diet | Regular physical activity | Moderate alcohol intake |
| --- | --- | --- | --- | --- | --- |
| No current smoking | 1 | -0.0113 | 0.0722 | 0.0022 | 0.0899 |
| BMI <30 kg/m^2^ |  | 1 | 0.0539 | 0.0591 | 0.0551 |
| Healthy diet |  |  | 1 | 0.0665 | 0.0230 |
| Regular physical activity |  |  |  | 1 | -0.0077 |
| Moderate alcohol intake |  |  |  |  | 1 |

Table S3: The lifestyle that included waist-to-hip ratio rather than BMI and risk of depression.

| Healthy lifestyle categories | Events/person-year | Incidence rate per 1000 person-year | Model 1 | Model 2 |
| --- | --- | --- | --- | --- |
| Unfavorable | 788/239729 | 3.29 (3.07-3.52) | 1 (Ref.) | 1 (Ref.) |
| Intermediate | 3350/1543158 | 2.17 (2.10-2.25) | 0.68 (0.62-0.73) | 0.67 (0.62-0.73) |
| Favorable | 1601/939434 | 1.70 (1.62-1.79) | 0.53 (0.48-0.58) | 0.52 (0.48-0.57) |
| *P* for trend |  |  | <0.001 | <0.001 |

Table S4: Risk of incident depression according to genetic risk and lifestyle stratified by sex and age.

|  | Sex | | Age | |
| --- | --- | --- | --- | --- |
|  | Male | Female | Age<60 | Age ≥60 |
| Genetic risk |  |  |  |  |
| Low | 1 (Ref.) | 1 (Ref.) | 1 (Ref.) | 1 (Ref.) |
| Intermediate | 1.03 (0.93-1.15) | 1.19 (1.09-1.29) | 1.08 (0.99-1.18) | 1.18 (1.07-1.30) |
| High | 1.16 (1.05-1.28) | 1.25 (1.16-1.36) | 1.22 (1.12-1.32) | 1.21 (1.10-1.34) |
| *P* for trend | <0.001 | <0.001 | <0.001 | <0.001 |
| Lifestyle |  |  |  |  |
| Unfavorable | 1 (Ref.) | 1 (Ref.) | 1 (Ref.) | 1 (Ref.) |
| Intermediate | 0.74 (0.64-0.85) | 0.70 (0.62-0.79) | 0.71 (0.63-0.80) | 0.75 (0.64-0.88) |
| Favorable | 0.54 (0.46-0.63) | 0.55 (0.48-0.63) | 0.53 (0.47-0.60) | 0.59 (0.50-0.70) |
| *P* for trend | <0.001 | <0.001 | <0.001 | <0.001 |

Table S5: Risk of incident depression according to genetic risk and lifestyle stratified by socioeconomic status.

|  | Socioeconomic status | | | *P* for interaction |
| --- | --- | --- | --- | --- |
|  | Low | Intermediate | High |  |
| Genetic risk |  |  |  | 0.013 |
| Low | 1 (Ref.) | 1 (Ref.) | 1 (Ref.) |  |
| Intermediate | 1.11 (0.98-1.27) | 1.26 (1.12-1.42) | 1.05 (0.95-1.16) |  |
| High | 1.35 (1.19-1.53) | 1.32 (1.17-1.48) | 1.08 (0.98-1.19) |  |
| Lifestyle factors |  |  |  | 0.001 |
| Unfavorable | 1 (Ref.) | 1 (Ref.) | 1 (Ref.) |  |
| Intermediate | 0.82 (0.65-1.05) | 0.76 (0.63-0.92) | 0.66 (0.59-0.75) |  |
| Favorable | 0.68 (0.53-0.87) | 0.59 (0.48-0.72) | 0.46 (0.40-0.53) |  |

Table S6: Genetic risk × lifestyle interactions.

|  | HR (95% CI) | *P* value |
| --- | --- | --- |
| Intermediate genetic risk × intermediate lifestyle | 0.94 (0.74-1.19) | 0.617 |
| Intermediate genetic risk × favorable lifestyle | 0.91 (0.71-1.17) | 0.463 |
| High genetic risk × intermediate lifestyle | 0.98 (0.77-1.24) | 0.856 |
| High genetic risk × favorable lifestyle | 1.03 (0.81-1.32) | 0.788 |

Interaction between the genetic risk and lifestyle for depression. Shown are the interaction effects between intermediate or favorable genetic risk and lifestyle compared to low genetic risk and unfavorable lifestyle.

Table S7: Risk of Incident depression According to Genetic and Lifestyle Risk after excluded first 2 years of developing depression.

| Subgroup | Hazard ratio  (95% CI) | P value |
| --- | --- | --- |
| Low genetic risk |  |  |
| Favorable lifestyle | 1 (Ref.) |  |
| Intermediate lifestyle | 1.32 (1.19-1.47) | 0.214 |
| Unfavorable lifestyle | 1.81 (1.49-2.21) | <0.001 |
| Intermediate genetic risk |  |  |
| Favorable lifestyle | 1.08 (0.96-1.21) | 0.017 |
| Intermediate lifestyle | 1.47 (1.33-1.64) | <0.001 |
| Unfavorable lifestyle | 2.19 (1.83-2.62) | <0.001 |
| High genetic risk |  |  |
| Favorable lifestyle | 1.25 (1.11-1.40) | <0.001 |
| Intermediate lifestyle | 1.54 (1.38-1.71) | <0.001 |
| Unfavorable lifestyle | 2.12 (1.77-2.53) | <0.001 |

Table S8: Association of genetic risk and lifestyle factors with incident depression using competing risk regression (Fine and Gray).

| Subgroup | Hazard ratio  (95% CI) | P value |
| --- | --- | --- |
| Low genetic risk |  |  |
| Favorable lifestyle | 1 (Ref.) |  |
| Intermediate lifestyle | 1.32 (1.19-1.46) | <0.001 |
| Unfavorable lifestyle | 1.77 (1.47-2.13) | <0.001 |
| Intermediate genetic risk |  |  |
| Favorable lifestyle | 1.10 (0.98-1.22) | 0.110 |
| Intermediate lifestyle | 1.49 (1.35-1.64) | <0.001 |
| Unfavorable lifestyle | 2.12 (1.79-2.51) | <0.001 |
| High genetic risk |  |  |
| Favorable lifestyle | 1.26 (1.13-1.40) | <0.001 |
| Intermediate lifestyle | 1.57 (1.42-1.73) | <0.001 |
| Unfavorable lifestyle | 2.15 (1.82-2.54) | <0.001 |

Fi
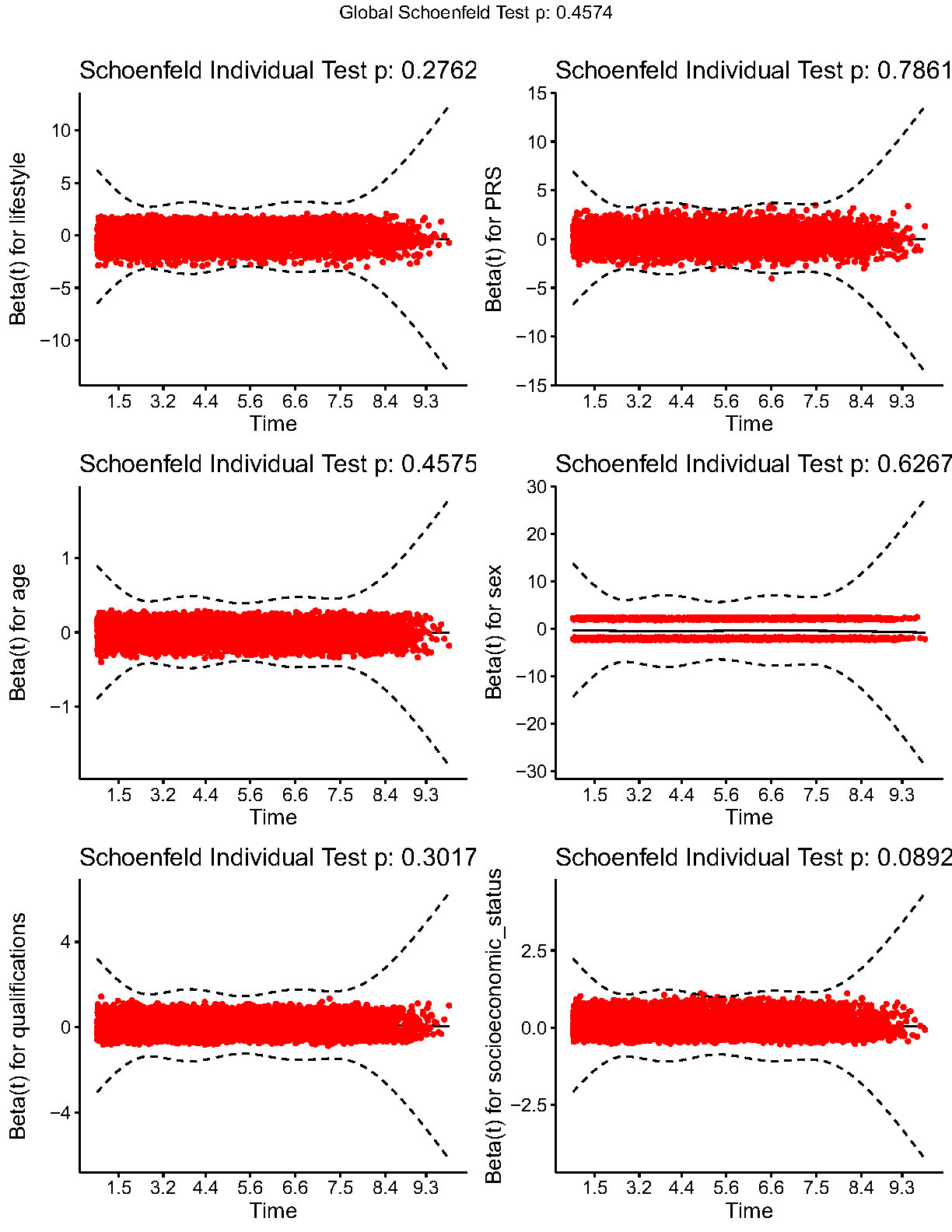
gure S1: Schoenfeld residuals plot of the proportional hazard assumption for Cox model.


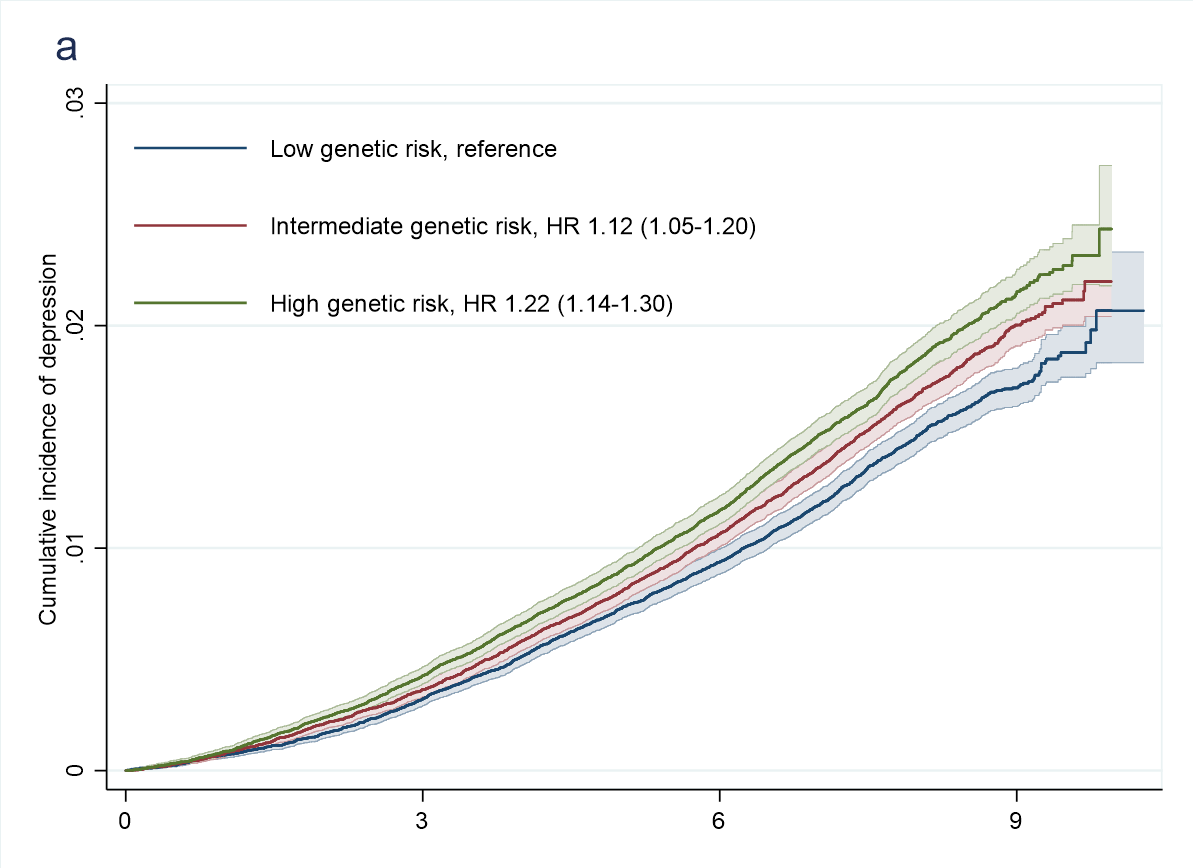

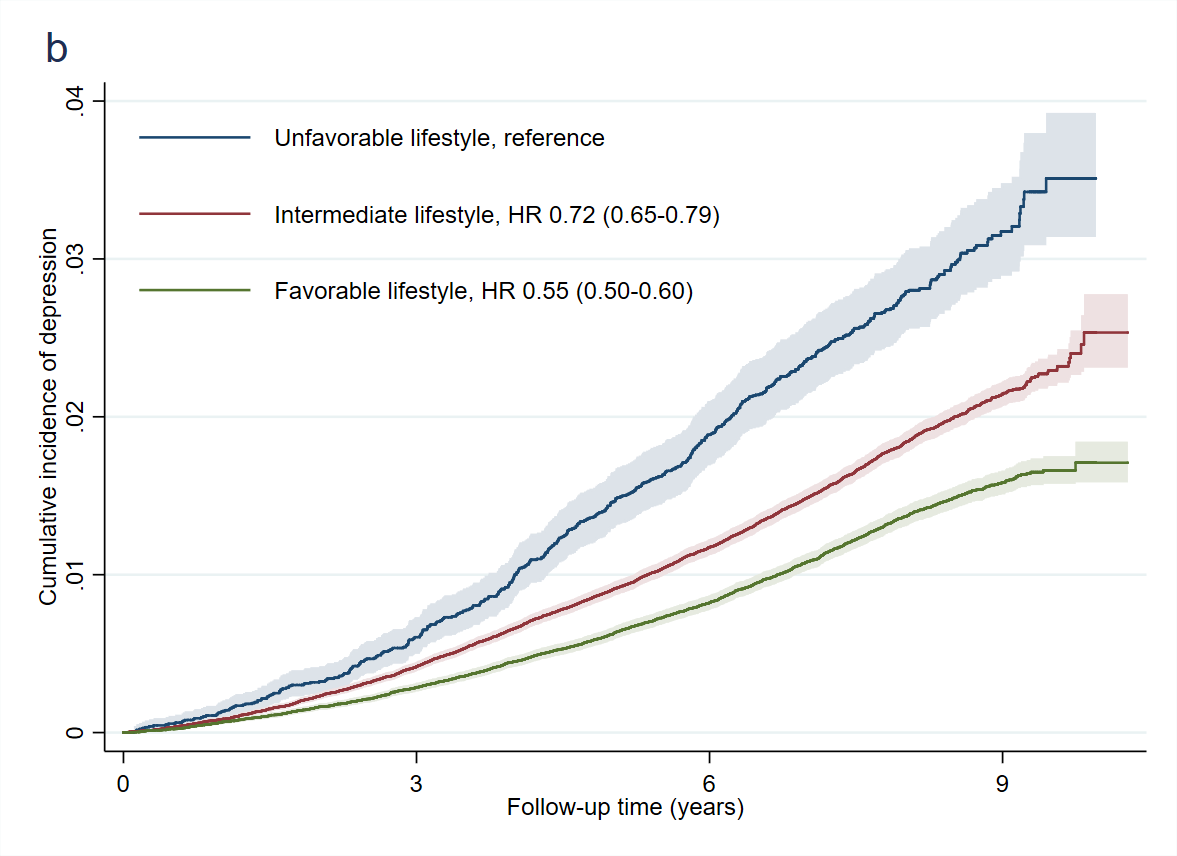
Figure S2: Cumulative incidence and hazard ratios of incident depression according to genetic risk and lifestyle factors profile. (a) Genetic risk and depression; (b) lifestyle factors and depression.


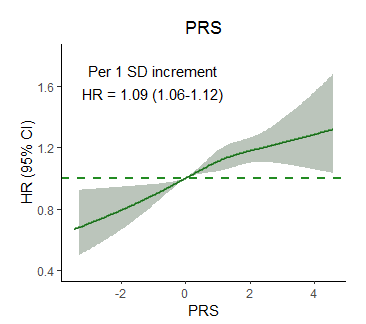
Figure S3: Dose-response association of continuous PRS with depression. P for non-linearity = 0.395.
